# Clinical and Structural Differences in Delusions Across Diagnoses: A Systematic Review

**DOI:** 10.3389/fnint.2021.726321

**Published:** 2022-01-24

**Authors:** Kelly Rootes-Murdy, David R. Goldsmith, Jessica A. Turner

**Affiliations:** ^1^Department of Psychology, Georgia State University, Atlanta, GA, United States; ^2^Tri-institutional Center for Translational Research in Neuroimaging and Data Science (TReNDS), Georgia State University, Georgia Institute of Technology and Emory University, Atlanta, GA, United States; ^3^Department of Psychiatry and Behavioral Sciences, Emory University School of Medicine, Atlanta, GA, United States

**Keywords:** delusion, transdiagnostic, structural, schizophrenia, Alzheimer's disease, Parkinson's disease, bipolar disorder

## Abstract

Delusions are marked, fixed beliefs that are incongruent with reality. Delusions, with comorbid hallucinations, are a hallmark of certain psychotic disorders (e.g., schizophrenia). Delusions can present transdiagnostically, in neurodegenerative (e.g., Alzheimer's disease and fronto-temporal dementia), nervous system disorders (e.g., Parkinson's disease) and across other psychiatric disorders (e.g., bipolar disorder). The burden of delusions is severe and understanding the heterogeneity of delusions may delineate a more valid nosology of not only psychiatric disorders but also neurodegenerative and nervous system disorders. We systematically reviewed structural neuroimaging studies reporting on delusions in four disorder types [schizophrenia (SZ), bipolar disorder (BP), Alzheimer's disease (AD), and Parkinson's disease (PD)] to provide a comprehensive overview of neural changes and clinical presentations associated with delusions. Twenty-eight eligible studies were identified. This review found delusions were most associated with gray matter reductions in the dorsolateral prefrontal cortex (SZ, BP, and AD), left claustrum (SZ and AD), hippocampus (SZ and AD), insula (SZ, BP, and AD), amygdala (SZ and BP), thalamus (SZ and AD), superior temporal gyrus (SZ, BP, and AD), and middle frontal gyrus (SZ, BP, AD, and PD). However, there was a great deal of variability in the findings of each disorder. There is some support for the current dopaminergic hypothesis of psychosis, but we also propose new hypotheses related to the belief formation network and cognitive biases. We also propose a standardization of assessments to aid future transdiagnostic study approaches. Future studies should explore the neural and biological underpinnings of delusions to hopefully, inform future treatment.

## Introduction

Delusions are a hallmark of schizophrenia and one of the main diagnostic criteria for the disorder (Maher, 2006; American Psychiatric Association, 2013). Delusions also present transdiagnostically, in neurodegenerative diseases, nervous system disorders, stroke patients, traumatic brain injuries, and other psychiatric disorders. There is even, albeit rare (current prevalence rate of 0.2%), a standalone diagnosis of delusional disorder that present solely with delusions and can maintain with reduced intensity or remit naturally (American Psychiatric Association, 2013; Opjordsmoen, 2014). Delusions are associated with increased caregiver burden, poorer medication adherence, and overall, worsening prognosis across disorders and can severely impact functioning and independent living (Ismail et al., 2011; Whitehead et al., 2012; Fischer and Sweet, 2016; Altamura et al., 2018; Warren et al., 2018).

Delusion type falls broadly into 12 different categories with some discrepancies: persecutory, jealousy, grandiosity, religious, delusion of reference, erotomania, guilt, somatic, and passive delusions such as, thought withdrawal, thought insertion, thought broadcasting, and the delusion of being controlled. There are additional categories of delusions that are more specific, such as Capgras delusion (believing family members are replaced by an identical imposter) and Othello's syndrome (delusional jealousy about family members) that have been observed across disorders (Moro et al., 2013). Different types of delusions may be associated with co-occurring symptoms (e.g., mood states) and overall clinical presentations that are etiologically heterogeneous. Furthermore, different types of delusions may indicate different underlying psychopathological constructs (e.g., deficits in self-monitoring vs. deficits in source monitoring) (Blakemore et al., 2000; Raveendran and Kumari, 2007; Corlett et al., 2010).

The assessments and measurements of delusional experiences depend largely on the primary diagnosis and delusional symptoms may vary in intensity, persistence, associated distress, and common themes or presentation types. The neurobiology that underlies this heterogeneity in delusions remains limited given obvious challenges in developing animal models of psychosis (Feifel and Shilling, 2010) although a recent mouse model has shown promising support for the involvement of the dopamine circuit in reality testing (Fry et al., 2020). Below, we will describe current theories of delusional development that have not been examined from a transdiagnostic standpoint. With differences in assessments, presentations, associated primary diagnoses, and lack of established animal models, the field would benefit from more clarity to better understand the etiology and neurobiology of delusions.

## Current Hypotheses on the Development of Delusions

### Delusions: The Dopamine Dysfunction Hypothesis

Psychosis has been shown to be associated with a dysfunction of the dopamine-dependent process of salience attribution (Kapur, 2003; Maia and Frank, 2017). Specifically, an increase in striatal dopamine synthesis capacity is related to psychosis progression (Howes et al., 2011). In schizophrenia, however, the disruption may be further explained by a combination of increased dopaminergic activity for irrelevant stimuli and a decrease in dopaminergic activity in regard to situation relevant stimuli (Maia and Frank, 2017). The mechanism of antipsychotic medications, mostly through dopamine (D2) antagonism (Li et al., 2016), suggests a causal relationship between psychosis and dopaminergic disruptions. However, it should be noted that this dopamine dysregulation model has mostly been developed in the context of psychosis in schizophrenia. Similarly in Alzheimer's disease, an excess of striatal dopamine D2/3 receptors was found to be related to delusion presence (Reeves et al., 2012). Levodopa (L-dopa), a precursor to dopamine, is the gold-standard medication for Parkinson's disease, and may result in the formation of delusions and hallucinations (Ruggieri et al., 1997; Swick and Walling, 2005). However, this association is not specific to delusions, such that there are remaining questions as to specificity of the relationship between dopamine dysregulation and delusions.

The mesolimbic pathway is a collection of dopaminergic neurons beginning at the ventral tegmental area in the midbrain and connecting to the ventral striatum (including the nucleus accumbens) of the basal ganglia in the forebrain. The release of dopamine into the nucleus accumbens regulates motivational salience, influences drive and behavior, and reward-related motor function learning (Kapur, 2003). Disruptions in the dopaminergic system may result in misread salient information, attention to irrelevant stimuli, and ultimately, disruptions to reward-related behavior (Kapur, 2003). Individuals with schizophrenia have been shown to assign overt salience to contextually irrelevant stimuli, potentially because of this disruption of dopamine release (Kapur et al., 2005). This disruption may explain the divergence of belief formation into psychosis formation. Therefore, this framework may begin to explain not only formation of delusions but hallucinations as well.

### Delusions: Deficits in Error Monitoring

Recently, delusions have been conceptualized as the result of defects in error monitoring, perhaps related to a disruption in dopaminergic pathways (Corlett et al., 2010; Krummenacher et al., 2010). Deficits in the ability to differentiate information-bearing patterns from noise result in noise taken in as salient information, also referred to as deficits in signal detection. Corlett and colleagues described this deficit as a two factor model (Corlett et al., 2010). First, the prediction error, or a discrepancy between the brain's prediction of a stimulus and the actual perception of that stimulus, occurs, and second, abnormal stimulus information is integrated into previous knowledge (Corlett et al., 2007, 2010). More specifically, the discrepancy between prediction and stimulus perception results in incorrect attention toward potential explanatory cues and subsequently, learning of misrepresentations of the environment, resulting in the formation of a delusion (Corlett et al., 2010; Corlett and Fletcher, 2015). Individuals with schizophrenia have been shown to have impaired error monitoring and importantly, defects in error awareness (Mathalon et al., 2002).

Prediction errors, in an inaccurate inference model for psychosis, have been associated with the right middle/inferior frontal gyrus (Griffiths et al., 2014). Previous structural studies have also implicated the left inferior frontal gyrus (IFG) in error monitoring (Mitchell et al., 2009; Sharot, 2011). As previously mentioned, the dopaminergic system also plays a role in signaling errors related to reward (or salience) prediction (Schultz and Dickinson, 2000). In functional studies, the anterior insula cortex and the anterior cingulate cortex are activated during errors in performance and error awareness (Klein et al., 2007; Ullsperger et al., 2010; Harsay et al., 2012). Specifically, the insula-cortico-thalamic circuit, including the dorsal and ventral areas of the anterior insula, is responsible for both error awareness and the processing of salience (Harsay et al., 2012). It remains unclear if this theory explains all delusion formation or relates only to delusions in patients with schizophrenia.

### Delusions: Cognitive Biases

Additional theories have been postulated about delusions being a form of cognitive bias. This theory states that the maintenance of delusional thinking requires a two-sided approach, or bias, to incoming information. There is a predilection for information supporting the delusion (confirmatory evidence), and an avoidance (or rejection) of evidence not supporting the delusion (non-confirmatory evidence) (Moritz and Woodward, 2006; Woodward et al., 2006). Specifically, cognitive biases such as jumping to conclusions, biases against disconfirming evidence (BADE), and liberal acceptance are more commonly seen in individuals with schizophrenia and delusions than healthy populations without psychosis (Moritz and Woodward, 2006; Veckenstedt et al., 2011).

Functional studies found the jumping-to-conclusion bias was associated with the dopaminergic reward system and the posterior cingulate cortex (Andreou et al., 2018). Bias against disconfirming evidence (BADE) was associated with increased visual network activity and reduced default mode network (DMN) activity when processing confirmatory evidence, and reduced activation in the orbitofrontal cortex, inferior frontal gyrus, and parietal cortex when processing disconfirming evidence in individuals with schizophrenia with delusional ideation (Lavigne et al., 2020).

These cognitive bias theories have all been suggested as separate explanations for the etiology of delusions. Together, the deficits in error monitoring (2.2) and cognitive biases (2.3) theories present the two main components of delusions, formation and maintenance. In other words, the delusion begins with an error in the processing of stimuli (a default) followed by avoiding the contradictory evidence while seeking out confirming evidence (a bias) to maintain the delusion. However, the theories have largely only been examined with individuals with schizophrenia or healthy controls using cognitive-based tasks (e.g., oddball task, antisaccade task) that represent circuits that underlie delusions. The use of these cognitive tasks is largely based on the limitations of examining active delusions in an MRI scanner and the previously mentioned lack of animal models. It remains unclear if these tasks activate all of the networks involved in delusion formation and maintenance. In addition, as the majority of studies examining the neurobiology and neuroanatomy of delusions focus on schizophrenia, it remains unclear if these theories of etiology and related circuitries generalize across the different diagnoses where delusions are present. Our review seeks to examine if these theories can explain the etiology of delusions as a whole or if they are explaining delusions within the context of a single disorder.

## Rationale for Review

For the purposes of this review, we sought to determine what contributions clinical phenotypes and structural neuroimaging have made to our understanding of the heterogeneity of delusions. The presence of delusions across different disorders suggests a potential common mechanism underlying the etiology of the symptom. Previous literature reviews have postulated hypotheses about the neurobiology underlying delusions but have yielded no transdiagnostic findings (Corlett et al., 2010). Previous neuroimaging studies have examined the neural correlates and gray matter changes associated with delusions (Whitford et al., 2009; Cascella et al., 2011; Ismail et al., 2011; Whitehead et al., 2012; Zhu et al., 2016). However, the majority of these neuroimaging studies examined delusions in only one disorder type. Even within a specific disorder, the identified neural substrates of delusions are not consistent across studies and not isolated within a brain region or known neural network. Examining the neural correlates associated with delusions across disorders could allow for identification of previously masked differences. Identification of gray matter atrophy or alterations in specific cortical regions (e.g., mesolimbic dopaminergic pathway) may also aid in supporting one of the above-mentioned models of psychosis and specifically delusions over another.

Studying delusions in their various clinical presentations, as opposed to being organized by the traditional diagnostic categories, might align more closely with the true underlying biology of the symptom. The focus of this paper was to review the current structural imaging research surrounding delusions across four diagnoses (schizophrenia, Alzheimer's disease, Parkinson's disease, and bipolar disorder) to examine to what extent the profile of delusional thought is clinically similar. We examined patient demographics (e.g., age), temporal development, and concurrent symptomatology that relate to the presence of delusions in these disorders to determine if any comorbid factors helped explain the etiology of delusions.

Examining the measurements utilized for delusions across disorders will allow for comparison of the presentation of the symptom itself as well as disorder-specific covariates. A review of disorder specific assessments is also detailed in Supplementary Appendix 1. We also reviewed structural brain imaging studies with the symptom of delusion to determine common neural underpinnings across disorders. We then compared our findings to the networks and brain structures previously implicated in the (1) current understanding of belief formation and (2) currently accepted theories on the formation and development of delusions.

There are hundreds of papers using structural imaging to study psychosis, but we will focus on the last decade of structural findings, specifically on delusions in the four identified disorders. The constrain to the four disorders is keeping with the currently available neuroimaging literature although we acknowledge that this review is not all-encompassing of disorders presenting with delusions. This review sought to add to the current literature from the last extensive review on the neurobiology of delusions (Corlett et al., 2010) and examine what additional brain structures within a multitude of disorders presenting with delusions have been identified in the last 10 years. The domain-level approach of this review may aid in the understanding of the etiology and maintenance of delusions and, ultimately, assist in treatment options for individuals presenting with delusions.

## Methods

**Figure 1:**
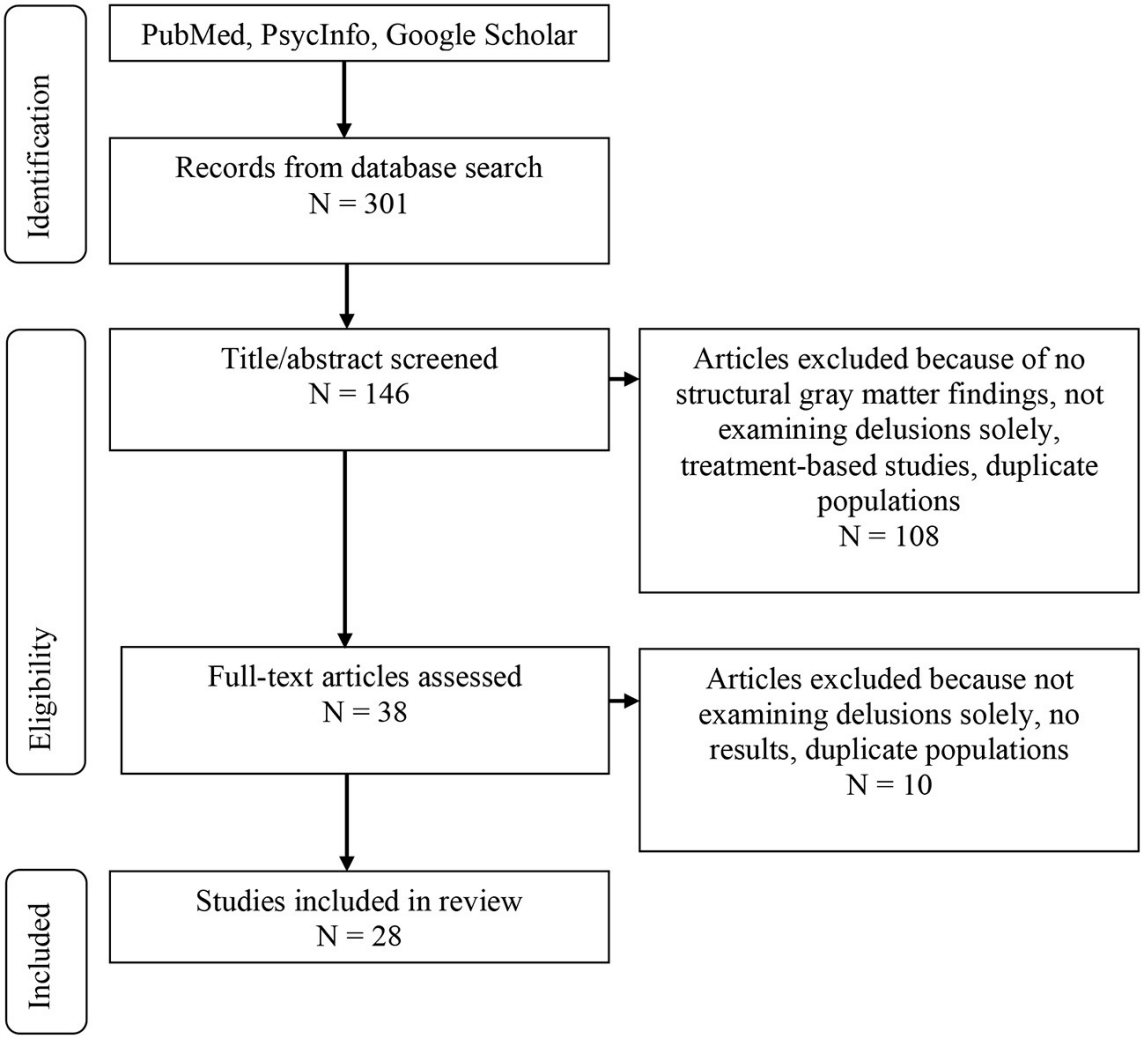
Literature review flow chart.

Electronic databases including PubMed, PsycInfo, and Google Scholar were searched for primary articles, meta-analyses, and case studies. Searches of the databases were performed using the keywords: [“delusion(s)” or “delusional” or “psychosis” or “psychotic features”] and [“assessment” or “measurement” or “diagnosis” or “structural” or “magnetic resonance imaging (MRI)” or “gray matter”] to find studies specifically assessing and/or reporting on structural neuroimaging of delusions. Abstracts and main texts were assessed with the following including/excluding criteria. The inclusion criteria for the neuroimaging articles were final publication dates from 2009 to 2020. Inclusion criteria for all articles included: examination of delusions in one of the following primary diagnoses such as schizophrenia, Alzheimer's disease, Parkinson's disease, or bipolar disorder; articles in peer-reviewed journals only; English language only; and in studies examining gray matter structural changes using MRI scanning. We allowed for neuroimaging studies that examined either case/control differences and/or dimensional studies (e.g., presence of delusions v. no delusions in the same disorder). Exclusion criteria were the following: publications including letters or brief communications; and studies that did not have results for delusions alone (e.g., examining psychosis as a combination of hallucinations and delusions only). Articles were rejected if it was determined from the title or abstract that the study did not meet the inclusion criteria. This search yielded a total of 301 studies, of which 155 were discarded based on the title/abstract. Of the 146 articles remaining, 118 were removed because of no structural gray matter findings, not examining delusions solely, not examining one of the four mentioned disorders, overlapping patient populations, or treatment-based studies. Twelve studies remained that discussed assessments and cognitive features in delusions and 16 neuroimaging studies remained. See Figure 1 for more details.

## Results

### Disorder Specific Presentations of Delusions

#### Schizophrenia

Schizophrenia (SZ) is a severe mental illness characterized by cognitive, behavioral, and emotional dysfunction (American Psychiatric Association, 2013). First-episode psychotic symptoms typically present in late teenage years through early to mid-thirties with men having, on average, a younger onset than women (American Psychiatric Association, 2013). Symptom presentation is often gradual in schizophrenia, but delusions are among the first symptoms to present and can diminish over time with some elderly patients reporting reduced or no significant delusions (American Psychiatric Association, 2013). As delusions are part of the diagnostic features in schizophrenia, they are very common, occurring in more than 90% of cases (American Psychiatric Association, 2013; Bebbington and Freeman, 2017). The most prevalent type of delusion is persecutory (more than 70% of first episode patients) but all other previously mentioned types have also been observed in schizophrenia (American Psychiatric Association, 2013; Coid et al., 2013; Picardi et al., 2018).

#### Bipolar Disorder

Bipolar disorder (BP) is a severe mood disorder characterized by extreme polar mood states, from depression to hypomania to mania (American Psychiatric Association, 2013). There are multiple forms of bipolar disorder defined mainly by the types of mood states; for example, Type I indicates fluctuation from depressed to manic states and Type II indicates fluctuations from depressed to hypomanic states (American Psychiatric Association, 2013). There are common neuroanatomical and genetic features that overlap between bipolar disorder and schizophrenia and even more overlap when further parsing bipolar disorder into bipolar disorder with psychotic features (Potash et al., 2003). Rate of psychosis among individuals with bipolar Type I disorder is 68 and 45% among those with bipolar Type II disorder (American Psychiatric Association, 2013). In terms of comorbid presentations, earlier age of bipolar onset is related to more psychotic features (Schürhoff et al., 2000). Delusions have also been noted in individuals with bipolar disorder independent of hallucinations or cognitive impairment (Tost et al., 2010).

Instances of hallucinations and delusions in bipolar disorder are most often mood congruent, indicating a relation to the current mood state of the individual (i.e., individuals in a depressed state have a higher prevalence of delusions of guilt) (Keck et al., 2003; Goodwin and Jamison, 2007). Delusions of grandiosity are most often related to individuals in a current manic state or mixed state (Keck et al., 2003; Goodwin and Jamison, 2007).

#### Alzheimer's Disease

Dementia is a neurodegenerative disease categorized by severe cognitive deficits including memory impairment and deficits in executive functioning (American Psychiatric Association, 2013). The most common type of dementia is Alzheimer's disease (AD), marked by memory impairment, difficulty concentrating, and visuo-spatial deficits. It is estimated that psychosis prevalence in Alzheimer's disease ranges from 10 to 73%, with an average of ~50% (Murray et al., 2014; Fischer and Sweet, 2016). Psychosis presentation in Alzheimer's disease is more common in the middle to later stages of the disease (Sweet et al., 2003). The clinical presentation may be completely free of psychosis for years prior to the first noted delusion (White and Cummings, 1996; Fischer and Sweet, 2016). However, delusions typically present earlier and more frequently than hallucinations in Alzheimer's disease (Cummings and Victoroff, 1990; Lopez et al., 1991).

In terms of comorbid symptoms, psychosis has been related to increased aggression, functional impairment, behavioral symptoms, rapid cognitive decline, mortality, and increased caregiver burden in individuals with Alzheimer's disease (Murray et al., 2014). Common delusions experienced in Alzheimer's disease are paranoid in nature; persecutory and fear driven (e.g., believing they are being stolen from) and delusions of misidentification like the previously mentioned Capgras delusion (Ismail et al., 2012).

#### Parkinson's Disease

Parkinson's disease (PD) is a neurological disorder that progressively affects motor movement. During the course of Parkinson's disease, neurons gradually break down causing a dopamine reduction that eventually results in abnormal brain activity and motor movements (Ravina et al., 2007; American Psychiatric Association, 2013). The typical age of onset for Parkinson's disease is 60 years old but there are also less common types of early onset and juvenile onset (American Psychiatric Association, 2013). Delusions occur in an estimated 5 to 16% of individuals with Parkinson's disease (Lee and Weintraub, 2012). As previously mentioned, there is also evidence of delusion onset presenting only after initiation of L-dopa treatment (Stefanis et al., 2010; Moroy et al., 2012; Moro et al., 2013).

Delusions in Parkinson's disease are markedly less common than hallucinations and are often assessed and treated together under the diagnosis of Parkinson's disease associated psychosis. A number of comorbid symptoms have been identified in Parkinson's disease associated psychosis; however, when examining only delusions, individuals tend to be younger and less likely to have cognitive impairment (Warren et al., 2018). The most common type of delusion in Parkinson's disease is paranoid in nature, similar to Alzheimer's disease. The themes of paranoid delusions broadly fall into the categories of persecution and jealousy. As individuals age, the most common theme is misidentification or Capgras syndrome.

### Neuroimaging Results

#### Schizophrenia

There were four studies that examined delusions in schizophrenia with a total sample of 198 individuals with schizophrenia and 126 healthy controls. One study examined only first episode psychosis (FEP) (Whitford et al., 2009), one study had a mix of patients with FEP and chronic schizophrenia (Zhu et al., 2016) and two studies examined only patients with chronic schizophrenia (Cascella et al., 2011; Spalletta et al., 2013). Two of the studies stated all individuals with schizophrenia were on at least one antipsychotic at the time of scan (Cascella et al., 2011; Spalletta et al., 2013) and two stated a mix of antipsychotic medication, antidepressant medication, and no medication at the time of scan (Whitford et al., 2009; Zhu et al., 2016). Three of the studies assessed delusions with the Positive and Negative Syndrome Scale (PANSS) (Whitford et al., 2009; Spalletta et al., 2013; Zhu et al., 2016) and one utilized the SAPS for delusion assessment (Cascella et al., 2011). The presence of delusions in schizophrenia was related to gray matter volume decreases in the left claustrum and right insula (Cascella et al., 2011), and in the dorsomedial prefrontal cortex (cluster = 54 voxels), centered on the medial frontal gyrus (Whitford et al., 2009). However, it was noted in the study on first episode psychosis (Whitford et al., 2009) that severity of delusions was positively correlated with dorsomedial prefrontal cortex volume. More specifically, excessive atrophy in the dorsomedial prefrontal cortex was related to less severe delusional formation (Whitford et al., 2009). Cascella and colleagues found delusion subscale scores were negatively correlated with gray matter volume in the left claustrum (cluster = 795 mm^3^) and right insula (cluster = 404 mm^3^) (Cascella et al., 2011). There was also reduced gray matter in the left insula (BA 47; cluster = 20 mm^3^) when comparing individuals with schizophrenia and delusions to those without delusions (Spalletta et al., 2013). However, Zhu and colleagues found that individuals with delusions showed greater gray matter volume in the right insula, superior temporal gyrus, and thalamus when compared to individuals with schizophrenia and no delusions; they were also not significantly different from healthy controls in these regions (Zhu et al., 2016). See Table 1 for more details. Three of the four studies examined the severity of delusions (Whitford et al., 2009; Cascella et al., 2011; Zhu et al., 2016) and three of the studies controlled for other positive symptoms that highly correlate with delusions (e.g., hallucinations) indicating that these results show structural changes, although varying across studies, that may be specific to delusions (Cascella et al., 2011; Spalletta et al., 2013; Zhu et al., 2016).

**Table 1:** Original structural imaging studies and phenotypes associated with delusions.

**Diagnosis**	**Study**	**Study type**	**Scan modality**	**Delusion type**	**N**	**Delusion assessment**	**Direction of findings**	**Imaging phenotype associated with delusions**	**Notes**
SZ	Cascella et al., 2011	Original	T1	All	SZ (43)	SAPS	↓ GM	L claustrum, R insula	70% D+
	Spalletta et al., 2013	Original	T1	Somatic	SZ (75); HC (75)	SAPS and PANSS	↓ GM	L insula	
	Whitford et al., 2009	Original	T1	All	FEP SZ (31); HC (21)	PANSS	↓ GM	DMPFC centered on medial frontal gyrus	Delusion severity positively correlated with GM
	Zhu et al., 2016	Original	T1	Paranoid (14), Disorganized (1), Undifferentiated (3), Residual (1)	SZ (49); D+ (19), D- (30); HC (30)	PANSS	D+ > D-; D+ < HC	L superior temporal gyrus, R insula, thalamus; amygdala, ACC	
BP	Radaelli et al., 2014	Original	T1	All	BP (73); D+ (34), D- (39)	Clinical notes	D+ < D-	Inferior frontal gyrus, insula, middle frontal gyrus	
	Tost et al., 2010	Original	T1	Persecutory delusions	BP (42); HC (42)	YMRS	Persecutory	L DLPFC, L medial PFC, inferior temporal lobe	
AD	Bruen et al., 2008	Original	T1	Misidentification	AD (31)	NPI	↓ in GM	R inferior frontal gyrus, inferior parietal lobule, inferior and medial frontal gyri, L claustrum	
AD	Fischer et al., 2016	Longitudinal	T1	All	MCI (7); AD (17)	NPI-Q	↓ in GM	L precuneus, insula, cerebellum, L superior temporal gyrus, L parahippocampal, R thalamus, R posterior cingulate	
	Graff-Radford et al., 2012	Original	T1	Othello syndrome	DLBD (5); AD (6); bvFTD (3); D-(14)	UPDRS-TD	D+ < D-	Dorsolateral frontal lobes, superior frontal gyri, R posterior lateral temporal lobe	
	Qian et al., 2019	Original	T1	Not specified	AD (59); D+ (23), D- (36)	NPI-Q	↓ in GM	Precentral and middle frontal gyri, SMA	Significant delusion X time interaction
	Serra et al., 2010	Original	T1	All	AD (27) MCI (19) HC (23)	NPI-12	↓ in GM	R hippocampus	5% D+
	Whitehead et al., 2012	Original	T1	Paranoid	AD (113); D+ (23)	NPI	↓ in GM	L medial orbitofrontal, L superior temporal, L insula	Results only in females
PD	Foley et al., 2017	5 case reports	MRI	Othello syndrome	5 case reports	Clinical notes		Normal brains (4), mild left fronto-temporal atrophy (1)	All delusions appeared following dopamine treatment
	Islam et al., 2015	Case report	MRI	Capgras delusion (animals and inanimate objects)	53 yo F	Clinical notes		Normal brain scan	Scan prior to delusion onset
	Mitchell et al., 2017	Review of case reports and one singular case report		Capgras delusion	15 case reports	None listed		Normal brains (2), mild frontotemporal atrophy (1), nil reported (8), cortical atrophy or microvascular disease (4)	
	Moroy et al., 2012	Case report	MRI	Olfactory delusion	59 yo F	Clinical notes		Normal brain scan	
	Sakai et al., 2019	Case report	Autopsy	Delusional jealousy	72 yo M	None listed		Mild frontal lobe atrophy	

*SZ, schizophrenia; FEP, first episode psychosis; AD, Alzheimer's disease; MCI, mild cognitive impairment; PD, Parkinson's disease; BP, bipolar disorder; PBD, psychotic bipolar disorder; NPBD, nonpsychotic bipolar disorder; HC, healthy controls; bvFTD, behavioral variant of Frontotemporal dementia; DLBD, dementia with Lewy body disease; GM, gray matter; MRI, magnetic resonance imaging; D+, delusions; D-, no delusions; UPDRS-TD, Unified Parkinson's Disease Rating Scale-Tremor Dominant; NPI, Neuropsychiatric Inventory; NPI-Q, Neuropsychiatric Inventory-Questionnaire; DIGS, Diagnostic Interview for Genetic Studies; MINI, Mini International Neuropsychiatric Interview; YMRS, Young Mania Rating Scale; PANSS, Positive and Negative Syndrome Scale; SAPS, Scale for the Assessment of Positive Symptoms; SCAN, Schedules for Clinical Assessment in Neuropsychiatry; CIDI-SF, World Health Organization Composite International Diagnostic Interview Short Form; L, left; R, right; F, female; M, male; SMA, sensorimotor area; DLPFC, dorsolateral prefrontal cortex; PFC, prefrontal cortex*.

#### Bipolar Disorder

There were only two studies that examined delusions alone in the context of bipolar disorder. There was a total sample of 115 individuals with delusions compared to 42 healthy controls and 39 individuals with bipolar disorder and no delusions. Both studies listed a total medication load, indicating individuals were prescribed either antipsychotics, antidepressants, mood stabilizers, anti-anxiety medications, a combination of the above, or medication naïve. One study had all individuals on antipsychotic medication at the time of scan and the other study had a mix of some individuals on antipsychotics and some medication naïve individuals. Decreases in gray matter were also found in the inferior frontal gyrus (BA 47; clusters = 141 mm^3^ and 17 mm^3^), insula (cluster = 83 mm^3^), and middle frontal gyrus (BA 9; cluster = 12 mm^3^) when compared to non-delusional bipolar individuals (Radaelli et al., 2014) and the inferior temporal lobe (cluster = 264 voxels) (Tost et al., 2010). Specific to persecutory delusions, there was a reduction in gray matter volume in the dorsolateral prefrontal cortex (three clusters with an average size of 5.8 voxels) (Tost et al., 2010). See Table 1 for further details.

#### Alzheimer's Disease

There were six studies with a total of 253 individuals diagnosed with Alzheimer's disease and delusions compared to 34 individuals with other dementias and 23 healthy controls included in this review. Across the six studies there was a mix of naïve antipsychotic medication users and individuals on antipsychotic medications (for varying durations). Cognitive status was added to the statistical models in four of the studies to confirm that the structural findings were specific to presence of delusions. Delusions were correlated with less gray matter in the right frontoparietal, left frontal lobe, right hippocampus, and the left claustrum (Bruen et al., 2008; Serra et al., 2010). In a longitudinal study, regional gray matter decreases were found in the insula (left cluster = 350 *k*; right cluster = 1180 *k*), precuneus (cluster = 3011 *k*), cerebellum (left cluster = 252 *k*; right cluster = 90 *k*), superior temporal gyrus (cluster = 902 *k*), right posterior cingulate (cluster = 74 *k*), thalamus (cluster = 200 *k*), and left parahippocampal gyrus (cluster = 633 *k*) in individuals who developed delusions (Fischer et al., 2016). In addition, there was also less gray matter in the sensorimotor area (BA 6; cluster = 8,904 mm^3^), left precentral gyrus (BA 6; cluster = 5,912 mm^3^), and frontal eye fields (BA 8; cluster = 3,440 mm^3^) in individuals with delusions and more accelerated atrophy in the temporal middle gyri (BA 20 and 21; clusters = 5,120 and 3,352 mm^3^, respectively) when compared to those without delusions (Qian et al., 2019). Specific to women with Alzheimer's disease and paranoid delusions, there was atrophy in the left lateral and medial orbitofrontal and superior temporal regions (Whitehead et al., 2012). Across multiple dementias (Alzheimer's disease, Lewy Body dementia, and frontotemporal dementia) there were gray matter reductions in the dorsolateral frontal lobes, specifically the superior frontal gyrus and the right posterior lateral temporal lobe (Graff-Radford et al., 2012). See Table 1 for more details.

#### Parkinson's Disease

There were a limited number of studies examining brain structures in individuals with Parkinson's disease and delusions. The studies identified in this review were three case reports and two meta-analyses consisting of 23 total case report findings. Two separate meta-analyses found both cases studies presenting with global brain atrophy or frontotemporal atrophy and then other case studies showing no changes related to delusions (Foley et al., 2017; Mitchell et al., 2017). Of note, these case studies had a combination of individuals experiencing both delusions and hallucinations with Capgras syndrome (Mitchell et al., 2017). Other case reports identified in this review found no significant brain abnormalities or changes in individuals with Parkinson's disease and delusions (Moroy et al., 2012; Islam et al., 2015). See Table 1 for more neuroimaging review results.

## Discussion

This paper reviews the current literature on clinical features, phenotypes, and neuroanatomical changes related to delusions across diagnoses where psychosis is common. The results show that the overall definition of delusions across disorders, although varying in prevalence and severity, consists of similar wording and description. Overall, the assessment and measurement of delusions demonstrates related, if not, exact overlap in the clinical definition of delusions across diagnoses; see Supplementary Appendix 1 for more details. All disorders refer to delusions as fixed, false, idiosyncratic beliefs that are inconsistent with reality and remain intact with contrary evidence presented. Therefore, we conclude that the overall symptom experienced across these disorders is largely the same.

Within each disorder, the symptom of delusions has different presentations. Primary types of delusions vary across diagnoses with persecutory delusions being most prominent in schizophrenia, Alzheimer's disease, and Parkinson's disease (Ismail et al., 2012; American Psychiatric Association, 2013; Picardi et al., 2018); Capgras syndrome and Othello's delusion most common in Alzheimer's disease and Parkinson's disease (Ismail et al., 2012; Foley et al., 2017; Mitchell et al., 2017); delusions of grandiosity more prominent in individuals with bipolar disorder in a manic state (Keck et al., 2003; Goodwin and Jamison, 2007); delusions of guilt more prominent in individuals in a depressed state (Keck et al., 2003; Goodwin and Jamison, 2007); and delusions of misidentification mostly associated with older cohorts regardless of diagnosis (Ismail et al., 2012; American Psychiatric Association, 2013; Foley et al., 2017; Mitchell et al., 2017; Picardi et al., 2018). Although prominent delusion types are noted in the literature, the vast majority of the assessments identified in this review did not contain questions related to the type of delusion. Expansion of the current assessments to include delusion type classification may aid in understanding the heterogeneous presentation of delusions in these disorders as specific delusion types appear to be more prominent in certain disorders, certain levels of cognitive functioning, age groups, and mood states. The findings of this review also strongly suggest that there should be more standardization of the assessment and measurement of delusions as there was little consistency in the assessments utilized across disorders.

In relation to age and clinical presentation, delusions vary inconsistently across disorders. There is no consistent age range for delusional onset in the four disorders. In schizophrenia, bipolar disorder, and some cases of Parkinson's disease, delusions are related to an earlier age of onset of the primary disorder. Delusions present as first or second order symptoms in individuals with schizophrenia (Maher, 2006; Whitford et al., 2009; American Psychiatric Association, 2013). Delusions are also present throughout the lifetime of individuals with schizophrenia with few reports of late life fading of persistent positive symptoms (American Psychiatric Association, 2013). The opposite trajectory is observed for individuals with Alzheimer's disease or Parkinson's disease. For individuals with Alzheimer's disease and Parkinson's disease, the clinical presentation may be completely free of psychosis for years prior to the first noted delusion (White and Cummings, 1996; Forsaa et al., 2010; Fischer and Sweet, 2016). In Alzheimer's disease, delusions are typically seen in the middle to late stages of the disorder. It is important to note that an earlier age of onset for Parkinson's disease is roughly 45–60 years old whereas with the psychiatric disorders, early onset is late teenage years to early twenties (American Psychiatric Association, 2013). Given this variation, delusion onset was also examined in the context of the disease course instead of age. The delusion onset related to the disease course still yields no significant overlap across the disorders as the same differences were seen (delusions in schizophrenia and Parkinson's disease are related to early stage, delusions in Alzheimer's disease are related to late-stage, and in bipolar disorder, delusion onset varied with in stages of the disease).

There are also no consistent comorbid symptoms or clinical presentations across the disorders. For individuals with Parkinson's disease, there is less cognitive impairment in those presenting with delusions (Warren et al., 2018) whereas delusions in Alzheimer's disease and schizophrenia are more likely to relate to cognitive deficits and overall, lower cognitive functioning (Sweet et al., 2003; American Psychiatric Association, 2013). Delusions in schizophrenia most commonly present with hallucinations and cognitive deficits (American Psychiatric Association, 2013). In Parkinson's disease, delusions are most highly associated with impulse control disorders and dopamine dysregulation syndrome (Warren et al., 2018). Delusions in bipolar disorder are most often reported during clinical mood states and change to be mood congruent (Goodwin and Jamison, 2007; Mahon et al., 2012). These findings are not surprising given the differences in clinical presentation between the disorders. Comorbid symptoms may relate more to the primary diagnosis and may not be biologically related to delusions. However, this hypothesis is limited based on the findings of this review and should be further examined in future studies.

In the neuroimaging studies, across diagnoses, the results showed varying degrees of alteration in the frontal and temporal regions across disorders. Although, there were some inconsistencies in the directionality of the gray matter alterations (Zhu et al., 2016), delusions were most associated with gray matter reductions in the dorsolateral prefrontal cortex (SZ, BP, and AD), left claustrum (SZ and AD), hippocampus (SZ and AD), insula (SZ, BP, and AD), amygdala (SZ and BP), thalamus (SZ and AD), superior temporal gyrus (SZ, BP, and AD), and middle frontal gyrus (SZ, BP, AD, and PD). The association of these additional findings to delusions may be explained when examining the function of these regions individually.

The claustrum has been linked to cognitive control, multi-sensory integration, consciousness, and task switching as well as cortically connected to the insula and the default mode network (Krimmel et al., 2019). The insula is involved with proprioception and the sense of self, self-awareness, more specifically, the posterior part of the insula is related to attention to and processing of salience (Craig, 2002; Harsay et al., 2012). As previously mentioned, the amygdala is connected with multiple regions of the brain and responsible for emotion regulation, emotional responsiveness, salience processing, as well as behavior modulation in connection to salient input and multiple neuromodulatory systems (e.g., dopaminergic) (Costafreda et al., 2008; Fadok et al., 2018). The hippocampus is primarily responsible for both short term and long term memory storage and retrieval (Squire, 1992) declarative memory, recollection of recognition memory, episodic memory, and familiarity (Brown and Aggleton, 2001; Kim, 2014; Bird, 2017) and along with the amygdala, salient information processing (Zheng et al., 2017). The posterior region of the superior temporal gyrus (specifically Wernicke's speech area, BA 22) is associated with auditory processing (Howard et al., 2000), and the caudal region relates to sentence comprehension (Hamilton et al., 2018). The thalamus serves as a relay station between internal and external information as well as is structurally related to the hippocampus, limbic system, and fornix. Specifically, the thalamo-cortical neurons are responsible for receiving external sensory information and relaying it upstream (Torrico and Munakomi, 2019) whereas the cortico-thalamo-cortical loop has been implicated in the maintenance of consciousness and attention to incoming visual stimuli (Trapp et al., 2012). However, these identified regions were not consistently reported across studies, nor across disorders presenting with delusions. In addition, the specificity of structural location (e.g., anterior vs. posterior insula) was not listed in a number of the studies. Since some of the overlap was between schizophrenia and bipolar disorder, those findings may be related to either the neural deterioration commonly seen in psychiatric disorders (DelBello et al., 2004; Lorenzetti et al., 2009; Kempton, 2011; Gupta et al., 2015; Torres et al., 2016), or the genetic and neural overlap amongst schizophrenia and bipolar disorder with psychosis (Tamminga et al., 2016), and less related to delusions specifically. Therefore, we conclude that the reductions in gray matter volumes identified in this review may not fully explain the development of delusional thinking.

The results of this review were then compared to structural regions mentioned in the other psychosis hypotheses (i.e., prediction error model, cognitive biases, and dopamine pathways as mechanisms of stimulus perception, information processing, and reward processing). There is some support for the prediction error model (Corlett and Fletcher, 2015) in the gray matter deficits (SZ, BP, and AD) in the dorsolateral prefrontal cortex (involved in expectation violation), insula (SZ, BP, and AD), and the middle frontal gyrus across all four disorders. These results also indicate some overlap with the mesolimbic dopaminergic pathway as there were structural alterations noted in the limbic system, specifically the amygdala, thalamus, and hippocampus. These areas have previously been implicated in salience prediction and importantly, errors in reward prediction. When taken with our previous hypothesis regarding delusions as negative prediction biases toward the environment, these results offer further support for an error in reward prediction, or aberrant salience, resulting in an observed negative environment. However, this theory is based only on partial results of this review as there are additional findings that do not fit into this prediction error model.

The mesolimbic dopaminergic pathway begins in the ventral tegmental area (VTA) in the midbrain, continues to the ventral striatum of the basal ganglia in the forebrain, and includes the nucleus accumbens. The neurons in the nucleus accumbens receive input from both the dopaminergic neurons of the VTA and the glutamatergic neurons of the hippocampus, amygdala, and the medial prefrontal cortex (mPFC) (Rubenstein and Rakic, 2013). Although gray matter reductions were found in the hippocampus, amygdala, and mPFC in this review, structural alterations were not consistently identified in the mesolimbic dopaminergic pathways. However, we caution that the lack of gray matter alterations in these pathways does not discredit the dopamine dysregulation hypothesis of psychosis. In fact, this review identified a number of studies in the Parkinson's disease literature that found delusions presenting only after treatment of levodopa (L-dopa) was started (Stefanis et al., 2010; Moroy et al., 2012; Moro et al., 2013) and that individuals with delusions were more likely to also have dopamine dysregulation syndrome (Warren et al., 2018). These dysregulations simply may not predict structural alterations.

As previously discussed, disruptions in the dopaminergic system may result in misread salient information or attention to irrelevant stimuli, and ultimately, disruptions in error processing, motivation salience (Kapur, 2003), and cognitive biases (e.g., jumping to conclusions). This dopamine dysregulation may be a downstream result of glutamate dysregulation in the prefrontal cortex (Stahl, 2018). Overactivation from the prefrontal cortex to the ventral tegmental area (VTA) from glutamate signaling may result in excess stimulation of the mesolimbic dopamine pathway. However, the results of this study do not clarify whether this disruption is an overproduction of dopamine, a combination of increased dopaminergic activity for irrelevant stimuli and a decrease in dopaminergic activity in regards to situation relevant stimuli (Maia and Frank, 2017) or follows an inverted U-shape, where both too much and too little dopamine result in a disruption (Cools and D'Esposito, 2011). The disruption may also be uneven throughout the cortex, with some dopaminergic loss in the striatum and then consequential “overdosing” in the intact structures (e.g., nucleus accumbens) (Cools et al., 2003). Moreover, it is also unclear where the disruption of dopamine occurs (e.g., dopamine synthesis vs. dopamine release). Regardless of the directionality, our results support that there are alterations in areas related to glutamatergic neurons that may be indirectly associated with the dopaminergic pathways. Together with the previous literature, we preliminarily hypothesize that these glutamatergic and related dopaminergic disruptions are the primary mechanisms behind delusions or at least, may explain the divergence of belief formation into psychosis formation. However, the exact relationship between dopamine and delusion formation needs to be examined further.

We then explored our findings in relation to the belief formation network as a potential explanation of delusion development. How individuals form beliefs is integral to understanding how delusions are formed as delusions are defined as fixed, false beliefs (American Psychiatric Association, 2013). Beliefs are formed from integration of previously learned and newly gathered information, and ultimately, guide decisions and actions (Bogousslavsky and Inglin, 2007; Rao et al., 2009). Self-reliant beliefs are most often positively biased and are adjusted to a greater extent when presented with new favorable information than when presented with new unfavorable information (Sharot and Garrett, 2016). This translates to a two-step process of information integration to update and maintain beliefs; (1) an increased tendency to alter beliefs in response to desirable information and (2) a reduced tendency, or an avoidance, to alter beliefs in response to undesirable information (Sharot and Garrett, 2016). These positive biases are most often observed in stress-free environments (Johnson and Fowler, 2011; Sharot and Garrett, 2016). The biases will revert (or reduce in optimism) to a more realistic bias in uncertain settings, stressful environments, or in the presence of harm (Johnson and Fowler, 2011; Sharot and Garrett, 2016). There are even reports of pessimistic and negative judgments in animal models resulting from stressful or negative treatment (e.g., dehorning, separation from mother) but overall, the results are inconsistent (Harding et al., 2004; Bateson and Matheson, 2007; Matheson et al., 2008; Bateson et al., 2011). Whether these beliefs invert completely to a negative bias in extreme stress conditions has yet to be fully examined in humans.

Previous functional imaging literature on belief formation showed incorporating previously held information with new belief formation was negatively correlated with activation in the bilateral inferior frontal gyrus (IFG) (Caton et al., 2020). Additionally, there was strong unilateral white matter connectivity between the left IFG and the multiple left subcortical regions (left amygdala, left hippocampus, left putamen, left pallidum, left thalamus, and insula) during integration of favorable information into new beliefs (Sharot, 2011; Moutsiana et al., 2015) and between the left IFG and the medial frontal cortex when estimating errors in good news (Sharot, 2011). Aligned with information integration, the left IFG [particularly Brodmann's areas (BA) 44, 45, and 47] is involved with speech processing and comprehension (Matchin et al., 2017), memory, attention to stimuli (Eliasova et al., 2014), evaluation of social interactions (Grecucci et al., 2013), and in combination with the temporal parietal junction (TPJ), creates a sensory motor loop for information coding (Downar et al., 2001; Johnson et al., 2019) and regulation of socially-induced emotions (Grecucci et al., 2013). Activation in the right TPJ and precuneus was also related to encoding event improbability based on previously received information (d'Acremont et al., 2013).

Therefore, we compared the identified regions of this review to those implicated in the belief formation network (left IFG, left amygdala, hippocampus, anterior putamen, pallidum, thalamus, and insula), and found some overlap with the regions implicated in schizophrenia, bipolar disorder, and Alzheimer's disease but less so with the regions implicated in Parkinson's disease. Interestingly, the belief formation network has been identified as purely unilateral in the left hemisphere whereas, our results showed structural changes in both hemispheres, but almost consistently unilateral in specific regions. The overlapping regions are associated with emotion and motivation (left amygdala, hippocampus, thalamus, and insula) (Costafreda et al., 2008; Li et al., 2011; Sharot, 2011; Huber et al., 2013) and are consistently reported in psychiatric disorders independent of delusions and therefore, may be more related to the primary disorder than the presence of delusions (DelBello et al., 2004; Lorenzetti et al., 2009; Kempton, 2011; Gupta et al., 2015; Torres et al., 2016).

Based on the findings of this review, we postulate that delusions are formed and maintained similarly to typical beliefs and that both aspects of delusional thinking (formation and maintenance) may be the result of disruptions in the dopaminergic circuit causing misrepresentations of salient and non-salient information, deficits in error processing, and ultimately, cognitive biases. Future research should utilize experimental paradigms in functional studies to isolate activated regions related to belief formation, error processing, and cognitive biases. In addition, future studies would benefit from examining delusions across diagnoses to narrow findings specifically related to delusion formation and not secondary to primary diagnoses.

### Limitations

There are some limitations in this review. In completing this review of the literature, there are a number of studies that mentioned the presentation of delusions in healthy cohorts, unipolar depression, right hemisphere stroke victims, following the development of brain lesions, and delusional disorder. This review was constrained to the four disorders because of a limited number of structural studies examining other disorders with delusions. Future studies should examine delusions across additional subsets of patient populations utilizing the NIMH Research Domain Criteria (RDoC) approach (RDoC; www.nimh.nih.gov/research-priorities/rdoc/index.shtml), especially given the limited studies on some groups (e.g., Parkinson's disease), to fully examine the neural underpinnings associated with delusions more generally. Future studies should also consider longitudinal approaches to parse out if the identified regions found in this review are causing delusions, or merely adding to the severity of presentation.

In addition, the direct comparison of the neuroimaging findings across diagnoses should only be accepted with serious consideration as there are some patient characteristics specific to each diagnosis that could impact how the findings relate to one another. For example, there were significant differences in the median ages of individuals with schizophrenia and bipolar disorder, and those diagnosed with Alzheimer's disease and Parkinson's disease. Although it should be noted that all of the structural findings between individuals with delusions and individuals without delusions (as opposed to those from longitudinal follow-ups) were either age-matched or did not have significantly differing means. Given the breadth of individuals and diagnoses reviewed, the potential confounds of medication (previous and current), length of illnesses, and treatment types (e.g., electroconvulsive therapy) also need to be taken into consideration in future imaging research that cuts across diagnoses. Of note, there were significant variations in the antipsychotic medications taken at the time of the MRI scan in all disorder categories. Some medications, specifically antipsychotic medication, have an effect on gray matter structures in the brain and these effects may be potentially masking or falsifying the relation of gray matter and delusions. Future studies may also consider a medication dosage by delusion interaction when examining structural differences.

There are some limitations within the neuroimaging studies that warrant further examination. A number of Alzheimer's disease and Parkinson's disease studies did not list the activation cluster coordinates and therefore, the regional description was taken at face value. Given the breadth of certain brain areas (e.g., thalamus, insula), the related diverse functions, and connections (e.g., thalamo-cortical vs. thalamo-cerebellar), exact activation coordinates may reveal more nuanced findings. Another limitation is the small sample size for the schizophrenia, Alzheimer's disease, and bipolar disorder studies. Specific to the Parkinson's disease literature, this review only found case reports (with a sample of one) and no sample-based imaging research studies. Future research would benefit from larger sample size and the related increased power to potentially unmask more associated regions. Lastly, this review was limited to structural neuroimaging studies. We limited our search in this way to focus on the anatomical or volumetric foundation in preparation of understanding the functional implications of delusions. Future studies should explore functional or circuitry studies using the structural bases from this review.

There are also a number of neuroimaging studies that consider delusions along a continuum, even finding differences between severe delusions and mild delusions. Although this review found evidence that delusions are not unidimensional, the scaling in most referenced assessments is narrow (see Supplementary Table 1) and therefore, the difference between mild and severe may be difficult to interpret. Finally, it should be noted that delusions are not typically a symptom experienced in isolation. Not all the studies in this review corrected for comorbid symptoms (i.e., hallucinations or cognitive deficits) and therefore, these results need to be taken with some consideration as there might be other symptoms contributing to the findings in certain regions. Even with these limitations, this review highlighted the shared symptom of delusions across disorders and the potential underlying biology that is associated with delusions.

### Future Directions

The results of this review support the need for a standardized assessment of delusions for all patient populations. Measurement consistency will aid the transdiagnostic approach to studying delusions and hopefully parse out subtle differences in the presentation (e.g., type) that have been mostly understudied in the current literature. As previously suggested, future studies would benefit from examining delusions in a multitude of the disorders they present in. This approach, along with simultaneous fMRI/Positron emission tomography (PET), may aid in measuring and relating the dysregulation of dopamine to brain regions associated with belief formation, cognitive biases, and hopefully, delusion formation.

### Conclusions

Delusions are defined globally as fixed, false beliefs that are incongruent with reality and are maintained even when the individual is presented with contrary evidence. There are some overlapping findings in gray matter effects in several brain regions, namely the dorsolateral prefrontal cortex, left claustrum, hippocampus, amygdala, insula, thalamus, superior temporal gyrus, and middle frontal gyrus, but for the most part, the findings showed a great deal of variability between the disorders. The findings of this review suggest that standardization of assessments would aid future transdiagnostic study approaches as there is currently no single instrument designed to be used across all disorders.

The results found that across disorders that present with delusions, alterations in gray matter were found in a variety of cortical areas, most not overlapping across disorders, and therefore, structural alterations are not fully explaining the development of delusions. The leading hypotheses of the neurobiology of delusions, namely the dysfunction of the mesolimbic dopaminergic pathway and aberration in how the brain computes and responses to prediction errors (Corlett et al., 2010), are only partially supported by this subsequent literature. There is not evidence that these entire networks are selectively affected structurally, leading to support for potentially different networks at play. In addition, the directionality of disruptions in dopaminergic pathways, as well as potential negative belief biases, and cognitive biases should be studied transdiagnostically to identify the neural and biological underpinnings of delusions and hopefully, inform future treatment of delusions.

## Data Availability Statement

The original contributions presented in the study are included in the article/Supplementary Material, further inquiries can be directed to the corresponding author/s.

## Author Contributions

KR-M completed the systematic review and wrote the manuscript. JT and DG reviewed and edited the manuscript and offered insights throughout the process. All authors contributed to the article and approved the submitted version.

## Conflict of Interest

The authors declare that the research was conducted in the absence of any commercial or financial relationships that could be construed as a potential conflict of interest.

## Publisher's Note

All claims expressed in this article are solely those of the authors and do not necessarily represent those of their affiliated organizations, or those of the publisher, the editors and the reviewers. Any product that may be evaluated in this article, or claim that may be made by its manufacturer, is not guaranteed or endorsed by the publisher.
