# Supplementary material for: Clinical and Structural Differences in Delusions Across Diagnoses: A Systematic Review
Source: Front Integr Neurosci. 2022 Jan 24;15:726321. doi: 10.3389/fnint.2021.726321 (PMC8818879; doi:10.3389/fnint.2021.726321)
Supplement: Supplementary file 1 [file Data_Sheet_1.DOCX]

**Supplemental Appendix 1**

***1.1 Assessments of Delusions***

The onset of delusions or hallucinations is an indicator of psychosis across disorders (American Psychiatric Association, 2013). In clinical settings, psychosis is regularly assessed regardless of diagnosis because of the implication for worsening condition or the necessity for changing medications. However, these assessments are typically covered in a clinical interview, most common are the Mini-International Neuropsychiatric Interview (MINI; Sheehan et al., 1998) or the Structured Clinical Interview for the DSM-IV (SCID; Spitzer, 1992), and generally encompass a broad range of symptoms, as psychosis is not always the sole concern. Therefore, there may be differences in the scope of assessment depending on the primary diagnosis and other patient-specific information (e.g., patient history). For example, discrepancies in the type of delusion are noted in the imaging research literature but not mentioned in most assessments or structure clinical interviews. Therefore, there is little research on the heterogeneity of delusion presentations.

This review sought to examine the current rating scales for psychosis in the four different disorders to determine consistency, overlap, and potential discrepancies in the presentation of delusions across disorders. We recognize the limitation that these symptoms specific assessments are more routinely utilized in research-based settings and less in clinical settings. For this review, the current standardized assessments and measurements for delusions frequently utilized in research-based settings were examined for the definition of delusions in each assessment, the questions related to delusions, how they were scaled, what aspects of delusions were assessed (e.g., duration of illness, type of delusion), and if there were any differences across the disorders.

**1.1.1 Schizophrenia.** Currently, the most commonly referenced scales for symptoms of schizophrenia are the Positive and Negative Syndrome Scale (PANSS), the Scales for the Assessment of Positive and Negative Symptoms (SAPS and SANS), and the Brief Psychiatric Rating Scale (BPRS) (Andreasen and Olsen, 1982; Kay et al., 1987). In schizophrenia, assessment of delusions involves measuring the severity of conviction, stability of delusion, and level of inappropriate behaviors related to the belief of the delusion (Andreasen & Olsen, 1982). In the PANSS, delusions are defined as “beliefs which are unfounded, unrealistic and idiosyncratic”. There is one item specifically related to delusions that states delusions as a “thought content expressed in the interview and [that has] influence on social relations and behavior” (Kay et al., 1987). There are other items that may also indicate delusional thinking, such as unusual thought content defined as “thinking characterized by strange, fantastic or bizarre ideas, ranging from those which are remote or atypical to those which are distorted, illogical and patently absurd” and guilty feelings defined as “sense of remorse or self-blame for real or imagined misdeeds in the past”. No further detail on type of delusion is described.

The SAPS is divided into four subscales: hallucinations, delusions, bizarre behavior, and positive formal thought disorder (Andreasen & Olsen, 1982). The subscale of delusions covers the 12 types of delusions previously mentioned. The scale details each type of delusion including examples of delusional thoughts and common behaviors to assess. For example, the delusion of jealousy listed examples such as “the person usually goes to great effort to prove the existence of the affair; searching for hair in the bedclothes, the odor of shaving lotion or smoke on clothing, or receipts or checks indicating a gift has been bought for the lover” (Andreasen & Olsen, 1982). The BPRS has 14 scale items related to psychotic symptoms, five of which are related to delusional presentation (i.e., grandiosity, guilt, suspiciousness, somatic concern, and unusual thought content) (Overall and Gorham, 1962). See Supplemental Table 1 for more details.

The PANSS, SAPS, and BPRS have high reliability with one another and are utilized in both clinical and research settings to evaluate delusions, the severity of presentation, the type of delusion, and the functional impairment in individuals with schizophrenia (Kumari et al., 2017). Given the reliability and validity of these scales, they have been adapted to study psychosis in other disorders (e.g., Parkinson’s disease) but these versions are not widely adopted (Voss et al., 2013).

**1.1.2 Bipolar Disorder.** The most noted scales for psychosis in bipolar disorder are the Young Mania Rating Scale (YMRS) and the previously mentioned Brief Psychiatric Rating Scale (BPRS) (Overall and Gorham, 1962; Young et al., 1978). The YMRS has one question, scaled zero to eight, regarding hallucinations and delusions together (Young et al., 1978). As previously mentioned, the BPRS has one question each for unusual thought content, somatic concerns, grandiosity, guilt, and an overall delusion question (Overall and Gorham, 1962). However, there is no delusion-specific or psychosis-specific assessment for bipolar disorder. The majority of studies examining bipolar disorder assessed the symptom of delusions using one of two structured clinical interviews; the Mini-International Neuropsychiatric Interview (MINI; Sheehan et al., 1998) or the Structured Clinical Interview for the DSM-IV (SCID; Spitzer, 1992), patient notes, and/or reports from family members (Altamura et al., 2018; Radaelli et al., 2014). These structured clinical interviews are not specific to bipolar disorder and are typically utilized for diagnostic purposes, not specific symptom assessment, in all psychiatric disorders. See Supplemental Table 1 for more details on the assessments.

**1.1.3 Alzheimer’s Disease.** There are a number of assessments developed to evaluate the psychiatric pathology that arises in Alzheimer’s disease. The majority of assessments did not ask the type of delusion experienced. A common assessment for psychosis utilized with individuals with Alzheimer’s disease is the Neuropsychiatric Interview (NPI) and the adapted, shortened version, the Neuropsychiatric Interview-Questionnaire (NPI-Q) (Cummings et al., 1994; Kaufer, 2000). The NPI script has nine yes/no questions regarding delusions, mostly referring to paranoid delusions or Capgras delusions (Cummings et al., 1994). The NPI-Q has one question regarding delusions, “Does the patient have false beliefs, such as thinking that others are stealing from him/her or planning to harm him/her in some way?” Another assessment used for psychosis is the caregiver-rated Behavioral Pathology in Alzheimer’s disease (BEHAVE-AD). There are seven questions regarding delusions, all referencing paranoid delusions (e.g., people are stealing things, delusion of abandonment). In general, if there was a question of delusion type on the assessment, it would only pertain to paranoia and Capgras syndrome. See Supplemental Table 1 for more details. In addition to the assessments listed, some studies identified in this review referenced delusions being assessed during in the clinical interview and from patient charts with no specific assessment.

Of note, assessment of psychosis in Alzheimer’s disease may be difficult because of the correlated cognitive impairments. Some studies theorized that the correlated cognitive impairment may cause individuals with Alzheimer’s disease and severe decline to be assessed as having hallucinations instead of delusions because of the impact on the individual’s ability to fully verbalize and describe the experienced delusion (Fischer and Sweet, 2016). If this theory is correct, there may be significant underreporting of delusional symptoms in this population.

**1.1.4 Parkinson’s Disease.** There are diagnostic criteria guidelines for psychosis, both delusions and hallucinations, in Parkinson’s disease based on the symptoms described by the Diagnostic and Statistical Manual for Mental Disorder IV-TR (DSM IV-TR) to help streamline the diagnosis of Parkinson’s disease associated psychosis (Ravina et al., 2007). Based on this review, the clinical use of this criteria does not appear to be widely adopted. Although the previously mentioned NPI, BPRS, PANSS, and SAPS have been validated for psychosis rating, it was noted that these scales are not ideal for clinical practice in this population given what some clinicians consider the unique presentation of psychosis in Parkinson’s disease (e.g., presenting after the primary diagnosis, not explained by a psychiatric disorder) (Fernandez et al., 2008). Based on this review, there is currently no globally utilized assessment for delusions in Parkinson’s disease.

There are two scales that were cited in studies of Parkinson’s disease associated psychosis, including the Movement Disorder Society Unified Parkinson Disease Rating Scale (MDS-UPDRS) and the adapted version of the Scale for Assessment of Positive Symptoms (SAPS-PD). The MDS-UPDRS includes one item related to thought disorder, a positive score on this item indicating presence of either hallucinations, delusions, or both. The SAPS-PD has been introduced in clinical trials for assessment of delusions in Parkinson’s disease although in completing this review, no current publication could be found that utilized the SAPS-PD in neuroimaging studies, although it is valid, sensitive to treatment changes, and easy to administer (Voss et al., 2013). This version uses the same wording and scaling system as the previously mentioned SAPS for schizophrenia (Andreasen, 1984). See Supplemental Table 1 for more details on assessments. In line with Alzheimer’s disease, there were a number of studies that referred to clinical patient notes for delusion identification (Graff-Radford et al., 2012; Warren et al., 2018).

| Supplemental Table 1. Assessments for Identifying Delusions Across Disorders | | | | | |
| --- | --- | --- | --- | --- | --- |
| Name of Assessment | **Related Disorder** | **Definition of Delusions** | **Number of Questions Related to Delusions** | **Scaling** | **Delusion Type** |
| Positive and Negative Symptom Scale (PANSS) | SZ | Beliefs which are unfounded, unrealistic and idiosyncratic | 6 | 1 to 7 | Overall delusions, grandiosity, suspiciousness/persecution, somatic, guilt, unusual thought content |
| Schedule for Assessment of Positive Symptoms (SAPS) | SZ | An abnormality in content of thought. False beliefs that cannot be explained on the basis of the patient’s cultural background. | 13 | 0 to 5 | Persecutory, jealousy, guilt, grandiose, religious, somatic, delusions of references, being controlled, mind reading, thought broadcasting, thought insertion, thought withdrawal, global rating |
| Brief Psychotic Rating Scale (BPRS) | SZ; BP | Unusual, odd, strange or bizarre thought content | 5 | 0 to 7 | Overall delusions, somatic, guilt, grandiosity, suspiciousness |
| Young Mania Rating Scale (YMRS) | BP | Content | 1 | 0 to 8 | Not specified; catchall for delusions and/or hallucinations |
| Neuropsychiatric Inventory (NPI) | AD | Beliefs that you [the rater] know are not true? | 1  (9 subquestions) | Frequency: 1 to 4; Severity: 1 to 3; Caregiver Distress: 0 to 5 | Persecutory, paranoia, Capgras syndrome, suspiciousness, television or magazine figures are in the home/talking and interacting with them |
| Neuropsychiatric Inventory Questionnaire (NPI-Q) | AD | Does the patient have false beliefs, such as thinking that others are stealing from him/her or planning to harm him/her in some way? | 1 | Severity: 1 to 3; Caregiver Distress: 0 to 5 | None specified |
| Name of Assessment | **Related Disorder** | **Definition of Delusions** | **Number of Questions Related to Delusions** | **Scaling** | **Delusion Type** |
| Behavioral Pathology in Alzheimer’s Disease (BEHAVE-AD) | AD | A false conviction, not a misidentification | 7 | 0 to 3 | One’s house is not one’s home, Capgras syndrome, delusion of abandonment, delusion of infidelity, non-paranoia |
| Movement Disorder Society-Unified Parkinson Disease Rating Scale (MDS-UPDRS) | PD | Thought disorder (due to dementia or drug intoxication) | 1 | 0 to 4 | Not specified; catchall for delusions and hallucinations |
| Scale for the Assessment of Positive Symptoms Adapted for Parkinson’s Disease (SAPS-PD) | PD | An abnormality in content of thought. False beliefs that cannot be explained on the basis of the patient’s cultural background | 4 | 0 to 5 | Persecutory, jealously, delusions of reference, and global delusions |

*Note*. SZ: Schizophrenia; BP: Bipolar Disorder; AD: Alzheimer’s disease; PD: Parkinson’s disease.
